# MiR-182-5p promotes the Metastasis and Epithelial-mesenchymal Transition in Non-small Cell Lung Cancer by Targeting EPAS1

**DOI:** 10.7150/jca.60419

**Published:** 2021-10-17

**Authors:** Wenxiao Yang, Yinan Yin, Ling Bi, Yichao Wang, Jialin Yao, Ling Xu, Lijing Jiao

**Affiliations:** 1 Department of Oncology, Yueyang Hospital of Integrated Traditional Chinese and Western Medicine, Shanghai University of Traditional Chinese Medicine, Shanghai, China.; 2 Cancer Institute of Traditional Chinese Medicine, Shanghai University of Traditional Chinese Medicine, Shanghai, China.; 3 Institute of Clinical Immunology, Yueyang Hospital of Integrated Traditional Chinese and Western Medicine, Shanghai University of Traditional Chinese Medicine, Shanghai, China.

**Keywords:** EPAS1, miR-182-5p, NSCLC, Metastasis, EMT

## Abstract

**Background:** Dysregulation of microRNAs (miRNAs) is associated with the pathogenesis of non-small cell lung cancer (NSCLC). However, the mechanisms through which miR-182-5p regulate NSCLC progression have not been established. This study aimed at evaluating the expression levels of miR-182-5p in human NSCLC and its function in lung cancer cells. Endothelial PAS Domain-containing protein 1 (EPAS1; also referred to as hypoxia-inducing factor 2A, HIF-2α) is a transcription factor that is responsible for induction of genes related to cell survival under hypoxia conditions. Hypoxia, an inherent feature of solid tumors, is associated with aggressive phenotypes, as well as resistance to radiotherapy and chemotherapy, which predict metastasis and poor prognosis.

**Methods:** The Cancer Genome Atlas (TCGA) dataset was used to investigate the association between miR-182-5p expression and clinicopathological characteristics as well as prognosis of NSCLC patients. Target genes of miR-182-5p were identified using the PITA, miRmap, microT, miRanda, PicTar, and TargetScan prediction tools. Transwell assays were performed to determine the potential functions of miR-182-5p in lung cancer cells. Luciferase reporter assays were performed to analyze regulation of the putative target of miR-182-5p while western blot assays were used to validate the luciferase results.

**Results:** miR-182-5p was found to be upregulated in NSCLC tissues and acted as an independent prognostic factor for tumor recurrence in NSCLC patients. Functionally, overexpression of miR-182-5p promoted lung cancer cell migration and invasion. Genome-wide gene expression analysis and luciferase report assays revealed that EPAS1 is a direct target of miR-182-5p. EPAS1 was negatively correlated with miR-182-5p expression in NSCLC tissues. Univariate and multivariate survival analyses identified EPAS1 as an independent prognostic factor for overall survival (OS) in NSCLC.

**Conclusions:** These findings imply that miR-182-5p promotes NSCLC progression by targeting EPAS1 and is, therefore, a potential indicator of tumor recurrence in NSCLC patients.

## Introduction

Globally, lung cancer is the leading cause of cancer-related mortalities 1. In 2018, China recorded an estimated 4.3 million new cancer cases and 2.9 million cancer-related mortalities 2. Over 80% of lung cancer incidences are NSCLC 3,4. High frequencies of recurrence and metastasis are highly associated with poor clinical outcomes of lung cancer patients. It has been postulated that miRNA deregulation is correlated with NSCLC progression 5. MiRNAs inhibit target mRNA based on total or partial complementarity by targeting the 3' untranslated region (UTR) of the mRNA 6. miR-182-5p, a member of the miR-183/96/182 cluster, is related to various cancers 7. Depending on cancer type, it can function as an oncogene or a tumor suppressor 8. Suppressed expression levels of miR-182-5p are associated with poor survival outcomes in neuroblastoma 9. However, miR-182-5p is involved in hepatocellular carcinoma (HCC) metastasis by targeting FOXO3a 10. While its overexpression promotes NSCLC metastasis by activating the NF-κB signaling pathway 11, 12. Moreover, upregulated miR-182-5p levels enhance drug resistance in cisplatin-treated A549 by regulating PDCD4 13.

The association between miR-182-5p expression and NSCLC recurrence has not been elucidated. In this study, the correlation between miR-182-5p and NSCLC recurrence among patients that had been previously subjected to curative surgery was investigated. Underlying mechanisms through which miR-182-5p promote NSCLC progression were also explored.

EPAS1 is a homolog of hypoxia-inducible factor 1α. It plays essential roles in cell survival and invasion through E-cadherin, Vimentin, Ki-67, CD31 14, and VEGFA 15 16. It has been associated with several human cancers 17. Studies have reported that EPAS1 is the primary target gene of miR-182-5p 18. However, its underlying mechanisms have not been established 18.

## Methods

### Clinical data

The microRNA-Seq data (513 cases) and the mRNA data (504 cases) and clinical information from The Cancer Genome Atlas (TCGA) database (https://genome-cancer.ucsc.edu; Apache-2.0 licence) were confirmed and downloaded as the study subjects. We enrolled patients with pathologically confirmed stage I-IV NSCLC. The cases were collected from several cancer centers in the United States according to strict standards, and the clinical information of patients was recorded and followed up in detail. Basic clinical information in the database included age, gender, pathological type, TNM stage (Table S1 & 2). GSE36681 (Normal 103 and Tumor 103) was downloaded from the Gene Expression Omnibus database (GEO, https://www.ncbi.nlm.nih.gov/geo/). GEO2R was used to determine the gene expression profiles of lung adenocarcinoma (LUAD) and normal control (NC) samples and identify genes that were differentially expressed. Target genes of miR-182-5p were identified using the PITA 19, miRmap 20, DIANA-microT 21, miRanda 22, PicTar 23, and TargetScan 24 prediction tools. Analysis of differentially expressed genes in NSCLC was performed using Gene Expression Profiling Interactive Analysis 2 (GEPIA2) 25. Correlation analysis between miR-182-5p and target genes was performed using ENCORI 26. Disease-free survival (DFS) was defined as the time between surgery and the first event of either disease recurrence or death from any cause. OS was defined as the time between the date of diagnosis and death from any cause.

### Cell culture

NSCLC cell lines (NCI-H1975, NCI-H460, A549, 95-D), human bronchial epithelial cells (16HBE) and human lung epithelial cell lines (BEAS-2B) were purchased from the Chinese Academy of Sciences Cell Bank. Cells were cultured in RPMI 1640 medium (Corning, USA) supplemented with 10% Fetal Bovine Serum (FBS) (Gibco, USA) and 100 units per ml of penicillin‐streptomycin solution. The 293T cells were cultured in Dulbecco's Modified Eagle Medium supplemented with 10% FBS (Gibco, USA). Incubation was performed in a humidified atmosphere containing 5% CO_2_ at 37 °C. Mycoplasma test of all cells was negative.

### miRNA and mRNA quantitative real-time PCR

Total RNA was extracted from NSCLC cells using the TRIzol reagent 27. Briefly, 2 µg of extracted RNA were reverse transcribed into complementary DNA (cDNA) with the application of reverse transcriptase (Thermo Fisher, USA). The qRT-PCR reactions were performed using Power SYBR Green PCR Master Mix (Kapa biosystems, USA), and GAPDH was used as the internal control. Then, miRNAs were isolated from total RNA using a High Pure miRNA isolation kit (TIANGEN, KR211-01, China) followed by their transcription through RT-PCR using a TaqMan MicroRNA Reverse Transcription kit (TIANGEN Technologies, FP411-01, China). Data were analyzed using the comparative Ct method (2-∆∆Ct). Three experiments were performed for each clone. Primer sequences used in this study are presented in the following information (Table 1 & 2).

### Invasion and migration assay

Transfected cells (2×10^4^) in 200 µl serum free medium were added to the upper compartment of chambers (Corning, NY, USA) which were pre-coated with Matrigel (Corning, NY, USA). Chambers were placed in 24-well plates with 0.5 ml of 15% FBS medium. After 24 h of incubation at 37 °C, chambers were obtained, the medium was discarded after which they were washed using PBS (5 times, each for 5 min). Immediately, chambers were fixed in 24-well plates containing 300 μl of 4% PFA for 30 min. Then, they were washed 3 times using PBS and stained with 300 μl crystal violet (0.1%) for another 90 min. Cells in the wells were washed using the PBS buffer. Then, cells in the upper compartment were wiped away while cells in the lower compartment were observed by light microscopy. The number of five high power fields per chamber were counted and mean value calculated. The migration assay was similarly performed as the invasion assay, except that no Matrigel was used.

### Western blot analysis

Cells were lysed using the RIPA buffer (Sangon, China) containing a Proteinase inhibitor (Roche, Switzerland) and a Pierce phosphatase inhibitor (Thermo Fisher, USA) 27. Protein extracts were boiled in a loading buffer followed by separation on a 15% sodium dodecyl sulfate-polyacrylamide gel electrophoresis platform (SDS-PAGE). Separated protein bands were transferred onto polyvinylidene fluoride membranes. Primary antibodies against EPAS1 (7096s, CST, USA), GAPDH (2118S, CST, USA), N-cadherin (13116S, CST, USA), Vimentin (5741S, CST, USA), E-cadherin (3195S, CST, USA), MMP-9 (13667S, CST, USA), MMP-2 (40994S, CST, USA), Snail (3879S, CST, USA), and Twist (69366S, CST, USA) were diluted at a ratio of 1:1000 in accordance with the manufacturer's instructions. Then, membranes were probed with a goat anti-rabbit IgG highly cross‐adsorbed secondary antibody (HSA0003, maibio, China) for 2 h at room temperature after which they were exposed to the enhanced chemiluminescence (ECL) reagent for visualization.

### Luciferase reporter assay

The 293T cells were cultured in 24-well plates and randomly allocated into four groups for transfection studies using the Lip3000 transfection kit (Thermo Fisher Scientific, USA). Plasmid sequences used in this study are shown in the following information (Table 3). For each group, co-transfection was performed using a control plasmid (40 ng), a recombinant plasmid (800 ng), and a final mimics concentration of 20 nmol/L. Each group was set with 5 multiple pores. Cells were lysed, after which positive transfection was detected using the double luciferase assay kit (Biology of Hanheng, China) after 48 h of transfection. After measuring using a chemiluminescence instrument, the blank hole's basal signal was subtracted from the data of each hole. The firefly fluorescence signal generated by the plasmid was used as the control. Relative fluorescence activities were divided by sea kidney's fluorescence signal and normalized for each group.

### Data analysis

Univariate Cox regression analysis was performed to identify primary prognostic factors while multivariate Cox regression analysis and construction of the K-M survival curve were performed to establish the risk score model, and identify independent prognostic factors. Receiver operating characteristic curve (ROC curve) analysis was performed to estimate the prognostic power of the risk score model. Results of the receiver operating characteristic curve (ROC) in IBM SPSS Statistics version 24.0 (SPSS Inc., Chicago, IL, USA) were used to determine the diagnostic critical value and obtain the AUC (Area under the curve). The Youden Index was calculated as: Youden index= sensitivity + specificity -1. The maximum value of the Youden index is the optimal miR-182-5p and EPAS1 bound value. Cut-off values under the ROC curve were grouped to determine the relationship between their expression levels and lung cancer prognosis. The GraphPad Prism version 7.0 (GraphPad Software, USA) was used to analyze data regarding the function of miR-182-5p, which were presented as means ± standard deviation (SD). Differences between groups were determined using the GraphPad Prism software. p≤ 0.05 was set as the threshold for statistical significance.

## Results

### miR-182-5p expression was associated with tumor recurrence in NSCLC

**Figure 1:**
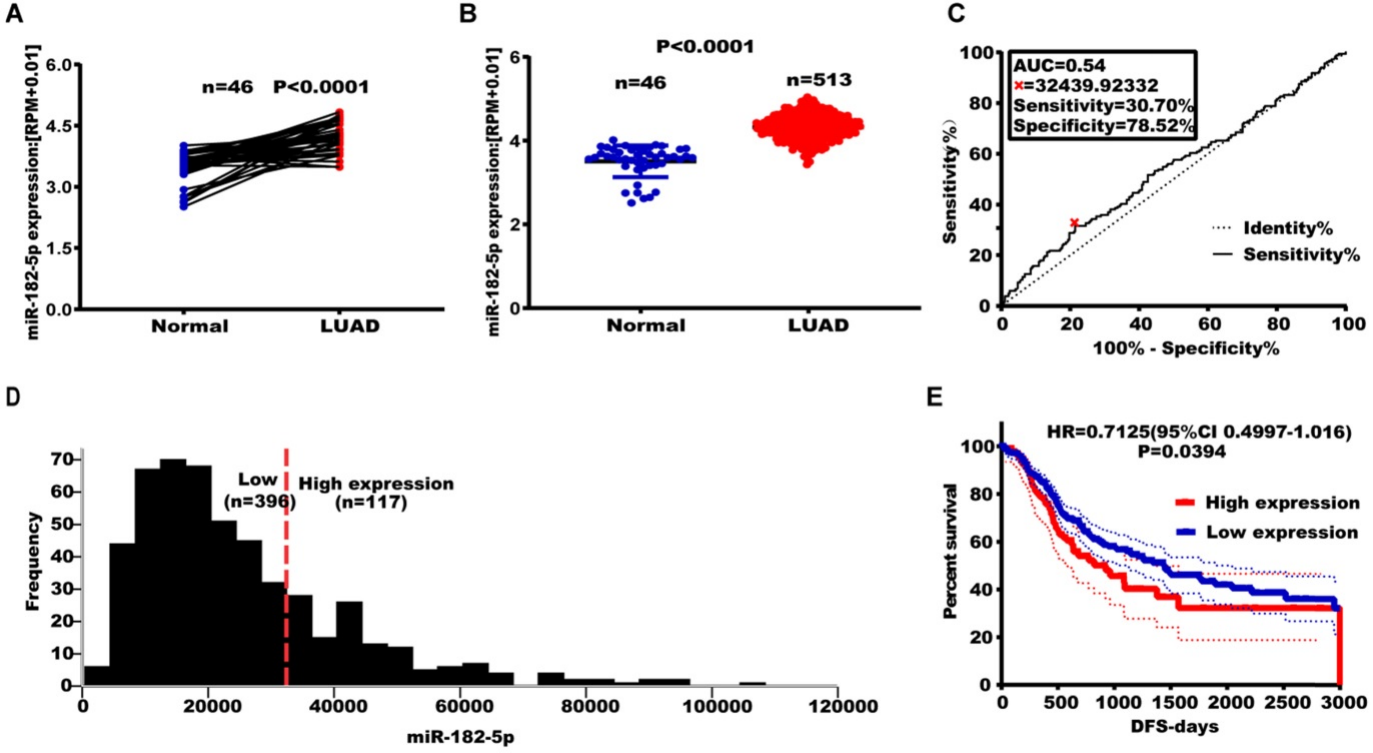
** TCGA analysis of expression levels of miR-182-5p in LUAD tissues. (A)** TCGA analysis of the expression levels of miR-182-5p in paired LUAD tissues. **(B)** TCGA analysis of the expression levels of miR-182-5p in unpaired LUAD tissues. **(C)** ROC curve used to obtain a cut-off value of miR-182-5p in LUAD. **(D)** LUAD patients categorized based on the cut-off value of miR-182-5p. **(E)** Kaplan-Meier analysis of the association between miR-182-5p expression and DFS.

The TCGA database was used to establish the relationship between miR-182-5p expression levels and tumor metastasis, recurrence, and long-term survival in lung adenocarcinoma patients. Among the 46 matched lung adenocarcinoma samples, expression levels of miR-182-5p in normal lung tissues were significantly elevated when compared to lung adenocarcinoma tissues (p<0.0001) (Figure 1A). Expression levels of miR-182-5p in lung tissues of 513 patients with unpaired lung adenocarcinoma (25183 ± 752.90) were significantly higher than those of 46 patients with normal lung tissues (p<0.0001) (Figure 1B). In addition, we downloaded the GSE36681 data from the GEO database for validation (p<0.0001, Figure S1). At the same time, in 45 matched samples of lung squamous cell carcinoma (LUSC) patients from the TCGA database, expression levels of miR-182-5p in normal lung tissues were significantly higher than those in lung squamous cell carcinoma tissues (p<0.0001; Figure S2). Expression levels of miR-182-5p in lung tissues of 478 unmatched patients with lung squamous cell carcinoma were significantly higher than those of 45 normal lung tissues (p<0.0001; Figure S3). Sensitivity and specificity of miR-182-5p were 30.7% and 78.52%, respectively, with a corresponding cut-off value of 32439.93 (Figure 1C & D).

Out of the 513 patients with lung adenocarcinoma, 396 patients (77.19%) were in the miR-182-5p low expression group (Figure 1D) and 117 patients (22.80%) were in the miR-182-5p high expression group. The DFS was 1447 days in the miR-182-5p low expression group versus 921 days. In the miR-182-5p high expression group (HR =0.7125, (95% CI: 0.4997-1.016; p= 0.0394) (Figure 1E). In the Cox univariate and multivariate regression analyses, T stage was established as a prognostic factor. The risk of recurrence and metastasis for T3 + T4 stages was 1.881 times higher than that for T1 + T2 stages (95% CI: 1.254-2.823; r = 0.0.632; p< 0.05). Meanwhile, the risk of recurrence and metastasis in lung adenocarcinoma patients with low expression levels of miR-182-5p was 0.691 times higher than that of patients with high expression levels (95% CI: 1.058-2.049). This finding suggests that miR-182-5p expression is an independent prognostic factor (r = 0.369; p<0.05; Table 4).

### miR-182-5p promotes NSCLC migration and invasion

**Figure 2:**
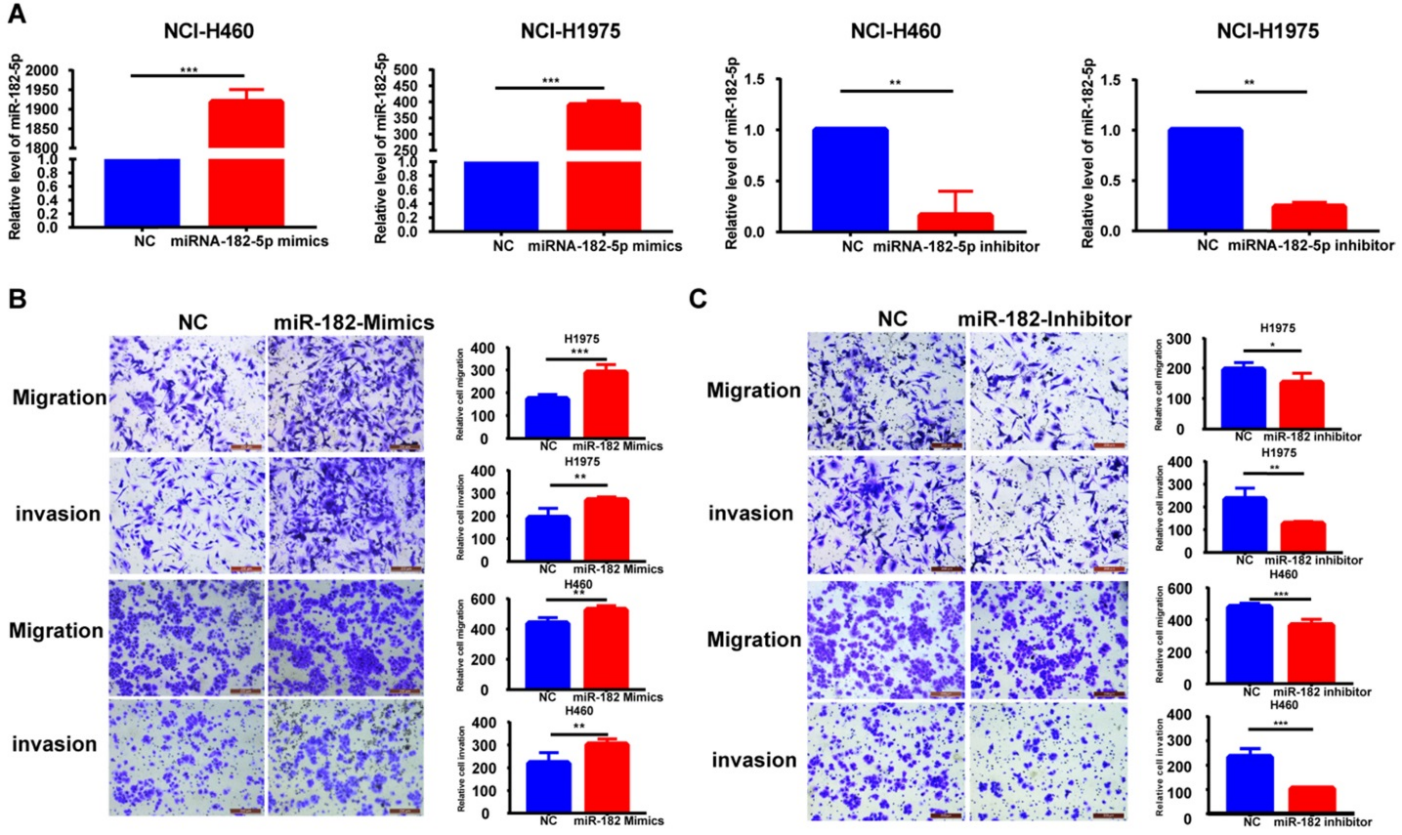
** Effect of altered miR-182-5p expression levels on NSCLC metastasis. (A)** Expression levels of miR-182-5p in different cell lines after transfection with miR-182-5p mimics/inhibitor. **(B)** Effect of miR-182-5p over-expression on lung cancer cell invasion and migration. **(C)** Effect of miR-182-5p under-expression on lung cancer cell invasion and migration. (*p<0.05, **p<0.01, ***p<0.001).

The expression of miR-182-5p was negatively correlated with tumor recurrence and metastasis. Therefore, we hypothesized that miR-182-5p inhibits NSCLC cell migration and invasion. Expression levels of miR-182-5p in commonly used human NSCLC cell lines and human lung epithelial cells were determined to test this hypothesis. Expression levels of miR-182-5p in 95-D, NCI-H1975, A549, and NCI-H460 cells were significantly elevated when compared to normal lung cells (16HBE and BEAS-2B) (p <0.05; Figure S4). Transfection efficiencies of miR-182-5p mimics/inhibitor in NCI-H1975 and NCI-H460 cell lines were confirmed by qRT-PCR (Figure 2A). NSCLC cell invasion and migration were promoted by elevated expression levels of miR-182-5p and inhibited by its suppressed expression (Figure 2B & C).

### Identification of EPAS1 as a direct target of miR-182-5p in NSCLC cells

**Figure 3:**
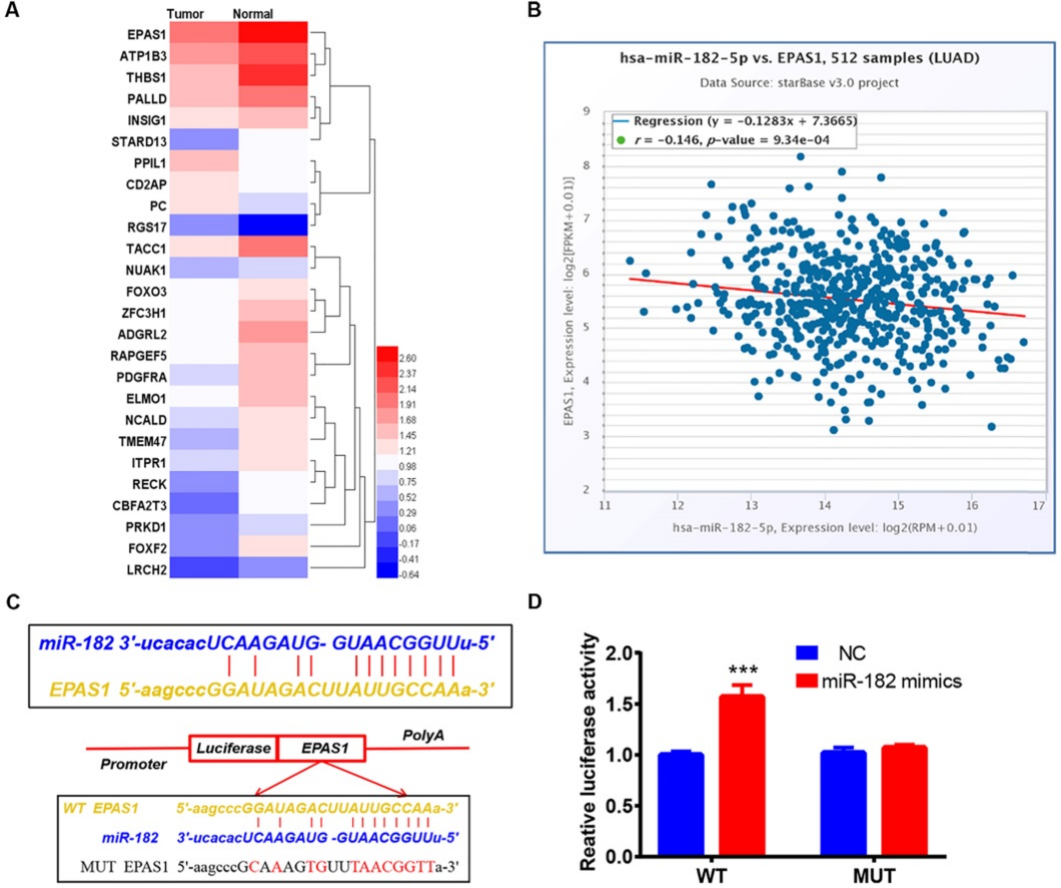
** Identification of EPAS1 as a direct target of miR-182-5p in NSCLC cells. (A)** Heatmap of 26 differentially expressed genes regulated by miR-182-5p in LUAD. **(B)** Pearson correlation analysis of the correlation between miR-182-5p and EPAS1 expressions in LUAD tissues. **(C)** Binding sites of miR-182-5p to WT or Mut 3' UTR of EPAS1. **(D)** Luciferase activities of WT or Mut 3' UTR of EPAS1 after co-transfection with miR-182-5p mimics and WT or Mut 3'UTR of EPAS1 in NSCLC cells. (***p<0.001).

Six different miRNA target prediction databases were used to select 121 common target genes to establish the molecular mechanisms involved in regulation of lung cancer cell migration and invasion by miR-182-5p (Table S3). Differentially expressed genes in lung adenocarcinoma were obtained from the TCGA project through GEPIA2 database analysis 25. There were 26 differentially expressed genes that were found to be regulated by miR-182-5p. Expression levels of EPAS1 in the gene list were significantly lower than those of adjacent tissues (Figure 3A). EPAS1 was negatively correlated with miR-182-5p expression in NSCLC tissues (r = -0.146, p = 0.000934; Figure 3B). Then, the Luciferase reporter assay revealed that wild type (WT) or mutant (Mut) 3'UTR of EPAS1 were successfully co-transfected with miR-182-5p mimics into the 293T cell line (Figure 3C). These findings imply that, compared to the miR-NC group, luciferase activity of the WT 3'UTR of EPAS1 was enhanced by increasing the expression of miR-182-5p. However, that of Mut 3'UTR of EPAS1 was unaffected by miR-182-5p's overexpression (Figure 3D). These findings confirm that EPAS1 is the target gene of miR-182-5p.

### EPAS1 expression is associated with poor survival in NSCLC

**Figure 4:**
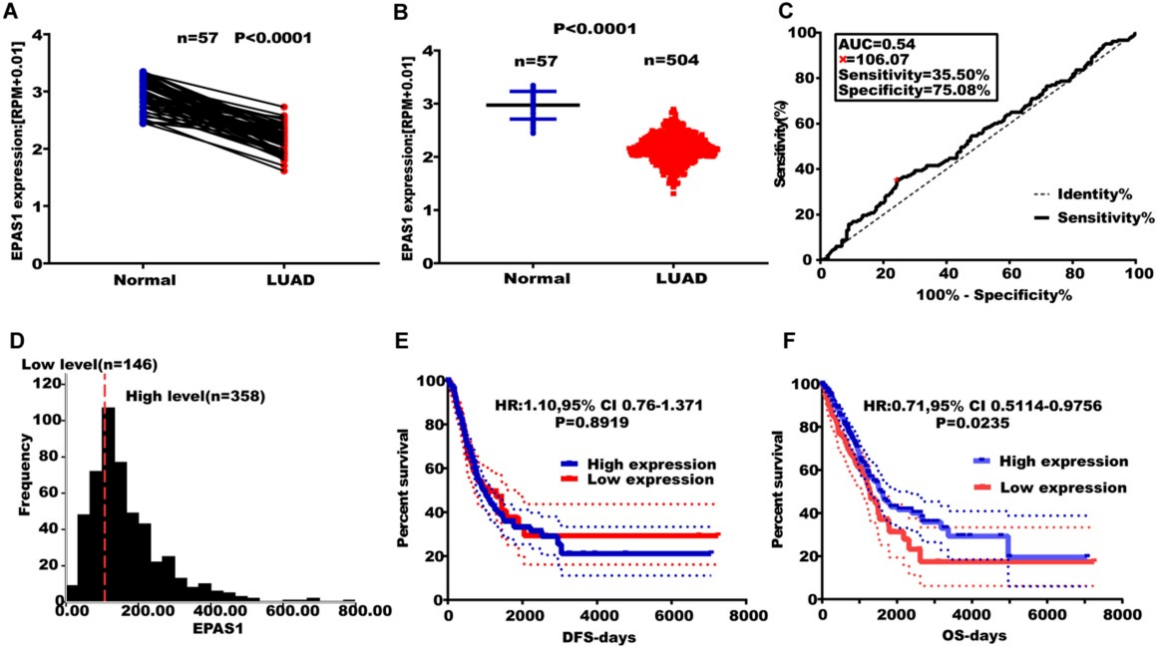
** Effect of altered EPAS1 expression levels on LUAD metastasis. (A)** TCGA analysis of the expression levels of EPAS1 in paired LUAD tissues. **(B)** TCGA analysis of the expression levels of EPAS1 in unpaired LUAD tissues. **(C)** ROC curve used to obtain a cut-off value of EPAS1 in LUAD. **(D)** LUAD patients, categorized based on the cut-off value of EPAS1. **(E)** Kaplan-Meier analysis of the association between EPAS1 expression and DFS as well as tumor recurrence. **(F)** Kaplan-Meier analysis of the association between EPAS1 expression and OS as well as poor survival.

EPAS1 expression levels were found to be suppressed in paired and unpaired NSCLC tissues (n = 513) compared to the 46 adjacent normal tissues (Figure 4A & B). An EPAS1 cut-off value was obtained using LUAD, based on OS time, survival status, and EPAS1 expression levels. Sensitivity and specificity of EPAS1 were 35.50% and 75.08%, respectively, with a corresponding cut-off value of 106.07 (Figure 4C & D). Based on the cut-off value, patients were grouped into the high and low-expression groups. Out of the 504 patients with lung adenocarcinoma, 358 patients were in the miR-182-5p low expression group, 146 patients were in the miR-182-5p high expression group (Figure 4D). The OS of the low expression group was not associated with age, gender, pathological stage, T stage, N stage, and M stage of patients (p > 0.05). Log-rank test revealed that patients with elevated EPAS1 expression levels resulted in a better overall survival outcome (p=0.0235). However, there were no significant differences in DFS for patients in both expression groups (p=0.8919) (Figure 4E & F). Cox univariate regression analysis revealed that clinical stage, T stage, N stage, and EPAS1 expression levels were the primary factors affecting lung adenocarcinoma recurrence and metastasis. The subsequent multivariate regression analysis revealed that T stage, N stage, and EPAS1 expression were independent prognostic factors for lung adenocarcinoma (p<0.05). The death risk for lung cancer patients with suppressed EPAS1 expression levels was found to be 1.478 times higher than that of patients with elevated EPAS1 expression levels (95% CI: 1.087-2.008; Table 5).

### miR-182-5p promotes epithelial-mesenchymal transition in NSCLC by targeting EPAS1

**Figure 5:**
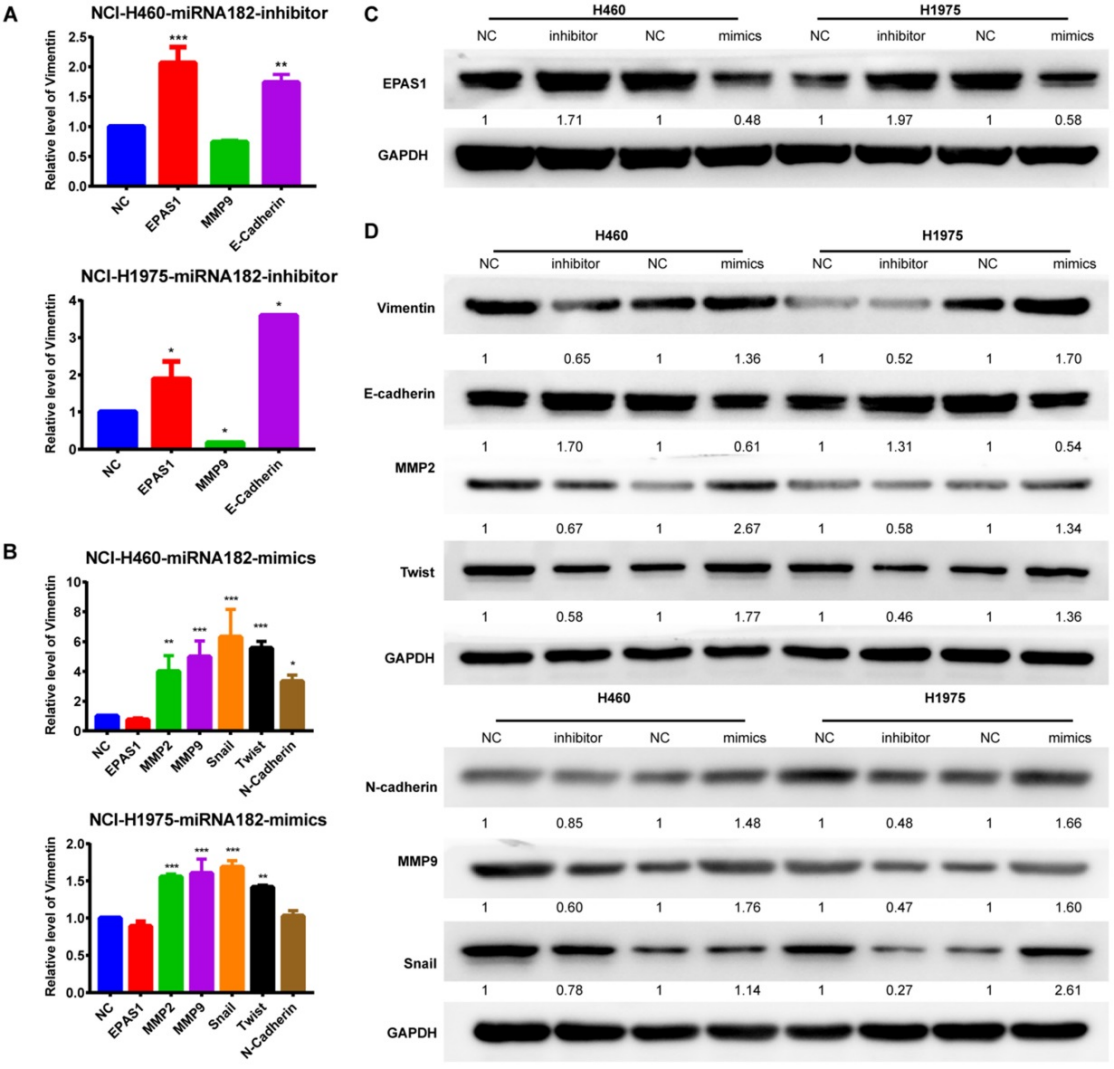
** EMT process and EPAS1 expression analyses by qRT-PCR and western blot. (A)** Effect of down-regulated miR-182-5p on the EMT process and EPAS1 expression in NCI-H460 and NCI-H1975 cells as determined by qRT-PCR. **(B)** Effect of overexpressed miR-182-5p on the EMT process and EPAS1 expression in NCI-H460 and NCI-H1975 cells as determined by qRT-PCR. **(C)** Effect of down-regulated and overexpressed miR-182-5p on EPAS1 expression in NCI-H460 and NCI-H1975 cells as determined by western blot. **(D)** Effect of down-regulated and overexpressed miR-182-5p on EMT expression in NCI-H460 and NCI-H1975 cells as determined by western blot. (*p<0.05, **p<0.01, ***p<0.001).

Both qRT-PCR and Western blot analyses revealed that overexpression of miR-182-5p suppressed the expression levels of EPAS1 in NCI-H1975 and NCI-H460 cells compared to the miR-NC group (Figure 5A & C). The EMT process was closely correlated with cancer cell migration and invasion. Quantification of mRNA expression levels of EMT-associated genes by qRT-PCR revealed that the epithelial marker, E-cadherin, was down-regulated. In contrast, the mesenchymal marker, MMP-9, was up-regulated in NCI-H460 and NCI-H1975 cells treated with a miR-182-5p inhibitor (Figure 5A). MMP-2, MMP-9, Snail, Twist, and N-cadherin were significantly up-regulated in NCI-H460 and NCI-H1975 cells treated with miR-182-5p mimics (Figure 5B). Inhibition of miR-182-5p suppressed protein expression levels of Vimentin, N-cadherin, MMP-2, MMP-9, Snail, and Twist but increased the expression levels of E-cadherin compared to the NC group in NCI-H460 and NCI-H1975 cells (Figure 5D). However, these changes were reversed by overexpression of miR-182-5p. These findings show that cells underwent the EMT process after transfection with miR-182-5p mimics/inhibitor.

## Discussion

Abnormal miRNA expression levels in early stages of lung cancer have been correlated with prognosis 28. Besides, miRNAs have a high pooled sensitivity and specificity in lung cancer diagnosis and treatment 29.

miR-182-5p is a small non-coding RNA that regulates gene expression by directly binding the 3'-UTR of the target mRNA. It regulates multiple biological processes by regulating over 1000 target genes involved in cell cycle, proliferation, apoptosis, metastasis, and metabolism 30-32. Moreover, it has been shown to promote the occurrence and development of breast cancer 33, lung cancer 34, thyroid cancer 35, medulloblastoma 36, and glioma 37, thereby leading to poor prognosis. It is usually highly expressed in lung cancer and tuberculosis patients 38. Some studies postulate that serum miR-182-5p is highly sensitive and specific as a potential biomarker for early lung cancer detection 28. Moreover, miR-182-5p has been shown to play a dual role in lung cancer development: as an oncogene in early stages of lung cancer and as an inhibitor of lung cancer metastasis in advanced lung cancer stages 34. miR-182-5p inhibits invasion and metastasis by regulating the EMT process through targeted down or up-regulation of ZEB2 (Zinc Finger E-Box Binding Homeobox 2), E-cadherin, and Vimentin proteins in NSCLC 39. Overexpression of miR-182-3p may inhibit EMT and metastasis by inactivating the Met/AKT/Snail pathway in NSCLC cells 40. Therefore, studies should aim at elucidating on the expression and function of miR-182 in lung cancer metastasis.

In this study, miR-182-5p expression was associated with age, pathological stage, and tumor size and is, therefore, a potential independent prognostic factor for tumor recurrence and metastasis in NSCLC patients. In addition, overexpression of miR-182-5p promoted lung cancer cell invasion and metastasis *in vitro*. Besides, miR-182-5p upregulated the expression of Twist, MMP-2 (matrix metallopeptidase 2), MMP-9 (matrix metallopeptidase 9), N-cadherin, Vimentin, and Snail proteins, which promoted the EMT process in lung cancer. Downstream target genes of miR-182-5p, predicted using an online platform, revealed that EPAS1, a potential target gene for miR-182-5p, affects cell invasion and migration. This finding was confirmed using the luciferase report assay.

EPAS1 is a transcription factor that is responsible for inducing genes associated with cell survival under hypoxic conditions 41. Studies have shown that EPAS1 promotes peritoneal carcinogenesis in NSCLC patients by enhancing MMT (mesothelial-mesenchymal transition), therefore, it is a potential prognostic marker or therapeutic target for NSCLC 17. Bioinformatics analysis of miR-182-5p revealed that EPAS1, PRKCE (protein kinase C epsilon), NR3C1 (nuclear receptor subfamily 3 group C member 1), and RHOB are the primary genes in the protein-protein interaction (PPI) network of lung squamous cell carcinoma 18. Extracellular ATP regulates EPAS1 and its target protein through the AKT-PGK1 pathway to inhibit breast cancer EMT 42. In this study, EPAS1 was negatively correlated with miR-182-5p expression. Elevated miR-182-5p levels significantly down-regulated EPAS1 expression *in vitro*. Therefore, miR-182-5p promotes lung cancer invasion and metastasis by negatively regulating EPAS1.

## Supplementary Material

Supplementary figures and tables.

## Figures and Tables

**Table 1:** Primer sequences for mRNA expression

Gene name		Sequence
E-cadherin	forward	CCTTGTGATCCGCCTGCCTTG
	reverse	CTGCCTGCCTGCCTTCTGATTAC
N-cadherin	forward	CAGAATCGTGTCTCAGGCTCCAAG
	reverse	CTGCGTTCCAGGCTGGTGTATG
Twist	forward	CGACGACAGCCTGAGCAACAG
	reverse	TCCTCGTAAGACTGCGGACTCC
Snail	forward	TTACCTTCCAGCAGCCCTAC
	reverse	CTTTCGAGCCTGGAGATCCT
MMP-2	forward	TTCCAAGTCTGGAGCGATGT
	reverse	CAGAAGCCGTACTTGCCATC
MMP-9	forward	GCCACTACTGTGCCTTTGAG
	reverse	TCAAAGACCGAGTCCAGCTT
Vimentin	forward	CGCCAACTACATCGACAAGG
	reverse	TGAAGCATCTCCTCCTGCAA
GAPDH	forward	CAGACCACAGTCCATGCCATCAC
	reverse	GACGCCTGCTTCACCACCTTC

**Table 2:** Sequences of the miR-182-5p mimic, inhibitor, and NC

Name	Sequences (5' to 3')
miR-182-5p NC	UUCUCCGAACGUGUCACGUTT
miR-182-5p mimic	UUUGGCAAUGGUAGAACUCACACU
miR-182-5p NC	CAGUACUUUUGUGUAGUACAA
miR-182-5p inhibitor	AGUGUGAGUUCUACCAUUGCCAAA

**Table 3:** Sequence of plasmids

Name	Sequence (5 'to 3')
miR-182-5p NC	UUCUCCGAACGUGUCACGUTT
	ACGUGACACGUUCGGAGAATT
miR-182-5p mimics	UUUGGCAAUGGUAGAACUCACACU
	UGUGAGUUCUACCAUUGCCAAAUU
EPAS1-WT-hsa-miR-182-5p	GGAUAGACUUAUUGCCAA
	UUGGCAAUG-GUAGAACU
EPAS1-MUT-hsa-miR-182-5p	GCAAAGTGUUTAACGGTT
	UUGGCAAUG-GUAGAACU

**Table 4:** Cox regression analysis of miR-182-5p expression as a tumor recurrence predictor

Variables	Univariate Cox's regression analysis		Multivariate Cox's regression analysis	
	RR (95%CI)	p-value	RR (95%CI)	p-value
**Age (years)**				
<65 versus >65	1.203 (0.891-1.6223)	0.227	NA	NA
**Gender**				
Male versus Female	0.953 (0.713-1.275)	0.746	NA	NA
**Pathological stage**				
I/IIIa versus IIIb+IV	1.385 (0.771-2.488)	0.276	NA	NA
**T stage**				
T1+T2 versus T3+T4	1.956 (1.305-2.931)	0.001	1.881 (1.254-2.823)	0.002
**N stage**				
Negative versus Positive	1.484 (1.103-1.995)	0.009	NA	NA
**M stage**				
Negative versus Positive	1.114 (0.812-1.528)	0.503	NA	NA
**miR-182-5p expression**				
High versus Low	0.711 (0.514-0.985)	0.040	0.691 (0.498-0.960)	0.028

RR: risk ratio; CI: confidence interval; NA: not analyzed.

**Table 5:** Cox regression analysis of prognostic factors for overall survival of lung adenocarcinoma

Variables	Univariate Cox's regression analysis		Multivariate Cox's regression analysis	
	RR (95%CI)	p-value	RR (95%CI)	p-value
**Age (years)**				
<65 versus >65	0.853 (0.633-1.148)	0.293	NA	NA
**Gender**				
Male versus Female	0.942 (0.704-1.260)	0.688	NA	NA
**Pathological stage**				
I/ IIIa versus IIIb+IV	2.171 (1.401-3.364)	0.001	NA	NA
**T stage**				
T1+T2 versus T3+T4	2.411 (1.666-3.487)	0.000	2.170 (1.493-3.154)	<0.001
**N stage**				
Negative versus Positive	2.550 (1.903-3.417)	0.000	2.298 (1.709-3.091)	<0.001
**M stage**				
Negative versus Positive	0.952 (0.847-1.070)	0.409	NA	NA
***EPAS1* expression**				
High versus Low	1.407 (1.038-1.906)	0.028	1.478 (1.087-2.008)	0.013

RR: risk ratio; CI: confidence interval; NA: not analyzed.
