# Supplementary material for: MiR-182-5p promotes the Metastasis and Epithelial-mesenchymal Transition in Non-small Cell Lung Cancer by Targeting EPAS1
Source: J Cancer. 2021 Oct 17;12(23):7120–9. doi: 10.7150/jca.60419 (PMC8558643; doi:10.7150/jca.60419)
Supplement: Supplementary file 1 — Supplementary figures and tables. [file jcav12p7120s1.pdf]

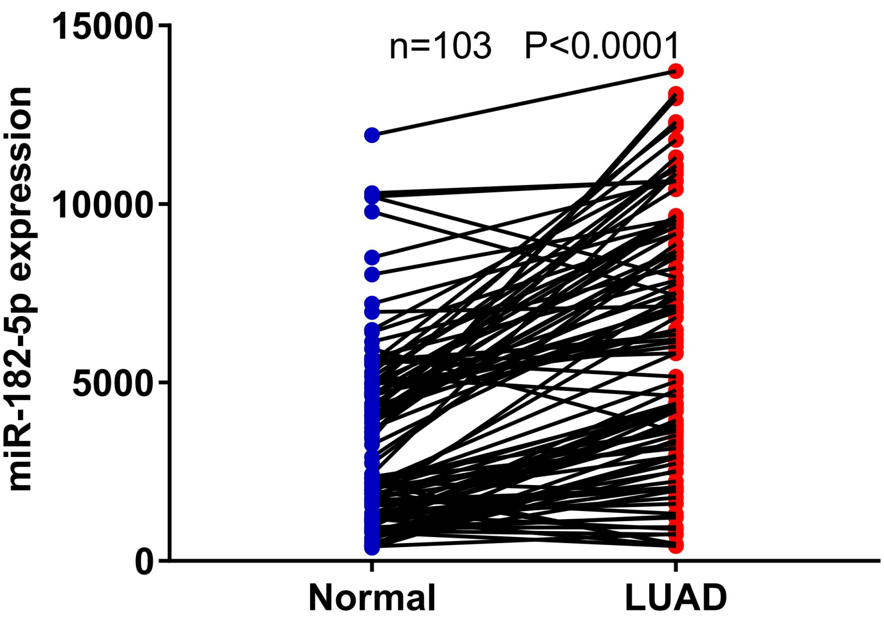

Figure S1. GEO analysis of the expression levels of miR-182-5p in paired NSCLC tissues.

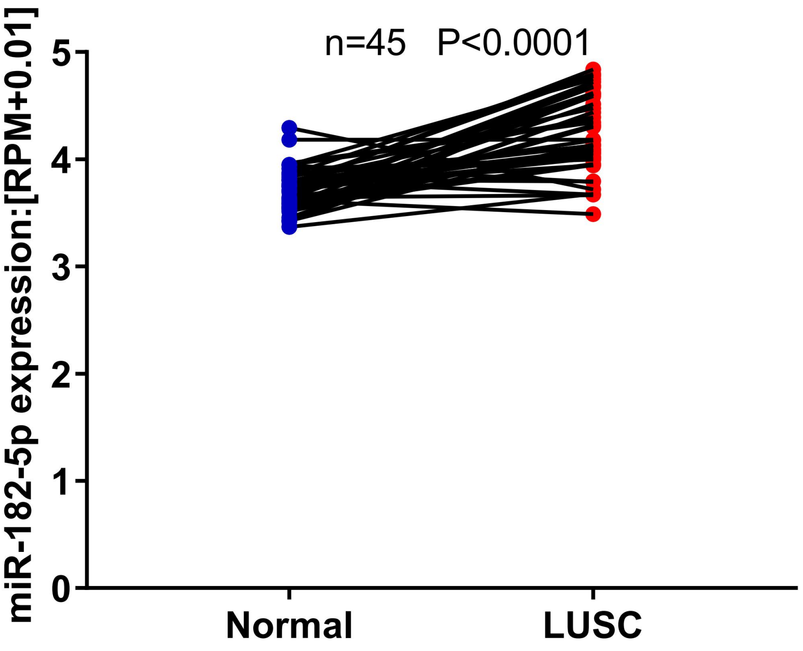

Figure S2. TCGA analysis of the expression levels of miR-182-5p in paired Lung squamous cell carcinoma tissues.

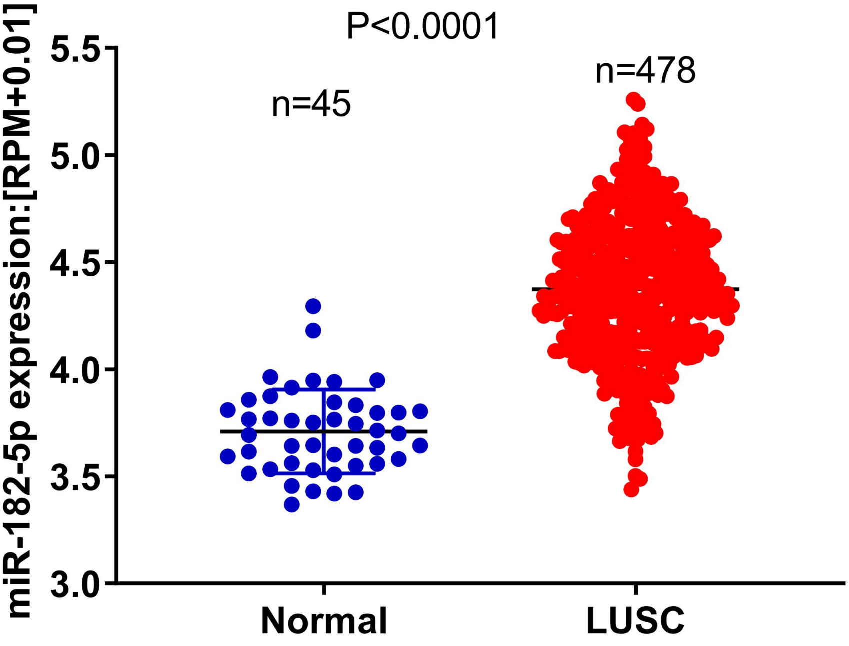

Figure S3. TCGA analysis of the expression levels of miR-182-5p in unpaired Lung squamous cell carcinoma tissues.

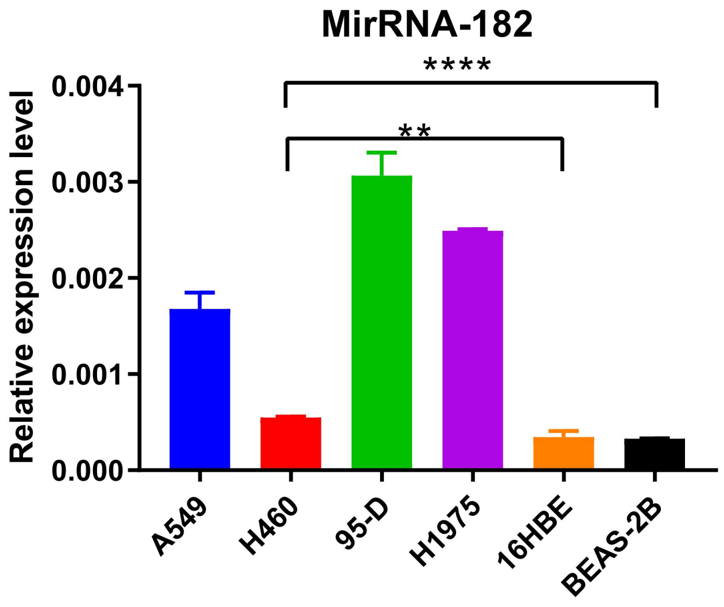

Figure S4. Expression levels of miR-182-5p in 95-D, H1975, A549, H460, 16HBE, BEAS-2B cells.(**p<0.01, ****p<0.001)

Table S1 The clinical information of Lung adenocarcinoma determined by the threshold value of miR-182-5p

Variables		Cases (N)	miR-182-5p		p value
			High expression	Low expression	
Total		513	117	396	
Age(years) *		494	113	381	0.236
	≥65	113	56（49.6）	163（42.8）	
	<65	381	57（50.4）	218（57.2）	
Gender					0.527
	Male	239（46.6）	51（43.6）	188（47.5）	
	Female	274（53.4）	66（56.4）	208（52.5）	
Pathological stage					0.536
	I/ IIIa	477（93.0）	107（91.5）	370（93.4）	
	IIIb+IV	36（7.0）	10（8.5）	26（6.6）	
T stage					0.647
	T1+T2	444（86.5）	103（88.0）	341（86.1）	
	T3+T4	69（13.5）	14（12.0）	55（13.9）	
N stage					0.743
	Negative	331（64.5）	74（63.2）	257（64.9）	
	Positive	182（35.5）	43（36.8）	139（35.1）	
M stage					0.653
	Negative	350（68.2）	82（70.1）	268（67.7）	
	Positive	163（31.8）	35（29.9）	128（32.3）	

* Note: Because of the original missing clinical information in the TCGA database, 494 cases of information were entered into the age distribution statistics, 19 cases were censored, with a censoring ratio of 3.70%; 4 cases were missing in the High expression group, with a censoring ratio of 3.42%; 15 cases were missing in the Low expression group, with a censoring ratio of 3.79%.

Table S2 The clinical information of Lung adenocarcinoma determined by the threshold value of EPAS1

Variables	Cases (N)	EPAS1		p value
		High expression	Low expression	
Total	504	358	146	
Age(years) *	494	350	144	1.000
≥65	221（44.6）	194（55.4）	80（55.6）	
<65	274（55.4）	156（44.6）	64（44.4）	
Gender				0.623
Male	234（46.4）	169（47.2）	65（44.5）	
Female	270（53.6）	189（52.8）	81（55.5）	
Pathological stage				0.269
I/ IIIa	466（92.5）	334（93.3）	132（90.4）	
IIIb+IV	38（7.5）	24（6.7）	14（9.6）	
T stage				0.773
T1+T2	437（86.7）	309（86.3）	128（87.7）	
T3+T4	67（13.3）	49（13.7）	18（12.3）	
N stage				0.538
Negative	326（64.7）	235（65.6）	91（62.3）	
Positive	178（35.3）	123（69.1）	55（37.7）	
M stage				0.418
Negative	340（67.5）	238（66.5）	102（69.9）	
Positive	25（5.0）	16（4.5）	9（6.2）	
Mx	139（27.6）	104（29.1）	35（24.0）	

*Note: Because of the original missing clinical information in the TCGA database, 494 cases of information were entered into the age distribution statistics, 10 cases were censored, with a censoring ratio of 1.94%; 8 cases were missing in the High expression group, with a censoring ratio of 2.23%; and 2 cases were missing in the Low expression group, with a censoring ratio of 1.37%.

Table S3: The 121 common target genes of miR-182-5p

Gene Name	Gene ID
IGSF3	ENSG00000143061
KHDC4	ENSG00000132680
CDC42BPA	ENSG00000143776
VAMP3	ENSG00000049245
AGO1	ENSG00000092847
ZMPSTE24	ENSG00000084073
ADGRL2	ENSG00000117114
PRKACB	ENSG00000142875
CAMSAP2	ENSG00000118200
FAM171A1	ENSG00000148468
SGMS1	ENSG00000198964
RHOBTB1	ENSG00000072422
JMJD1C	ENSG00000171988
KIAA1217	ENSG00000120549
SLC35G1	ENSG00000176273
ADD3	ENSG00000148700
PC	ENSG00000173599
CTTN	ENSG00000085733
CUL5	ENSG00000166266
KDM5A	ENSG00000073614
GXYLT1	ENSG00000151233
RARG	ENSG00000172819
ZFC3H1	ENSG00000133858
EEA1	ENSG00000102189
NUAK1	ENSG00000074590
AEBP2	ENSG00000139154
FRS2	ENSG00000166225
DENR	ENSG00000139726
STARD13	ENSG00000133121
INTS6	ENSG00000102786
PCDH8	ENSG00000136099

MBNL2	ENSG00000139793
ABHD13	ENSG00000139826
PRKD1	ENSG00000184304
GMFB	ENSG00000197045
ZFP36L1	ENSG00000185650
KTN1	ENSG00000126777
SLC39A9	ENSG00000029364
PCNX1	ENSG00000100731
ADAM10	ENSG00000137845
THBS1	ENSG00000137801
SNAP23	ENSG00000092531
CBFA2T3	ENSG00000129993
CHMP1A	ENSG00000131165
ZNRF1	ENSG00000186187
CDC27	ENSG00000004897
MMD	ENSG00000108960
ARHGDIA	ENSG00000141522
KCTD2	ENSG00000180901
ELL	ENSG00000105656
TMEM145	ENSG00000167619
BCL2L12	ENSG00000126453
PREPL	ENSG00000138078
RTN4	ENSG00000115310
PPP3R1	ENSG00000221823
SP3	ENSG00000172845
NCKAP1	ENSG00000061676
KLF7	ENSG00000118263
RAB10	ENSG00000084733
BABAM2	ENSG00000158019
EPAS1	ENSG00000116016
KCMF1	ENSG00000176407
TMEM50B	ENSG00000142188
DSCAM	ENSG00000171587
ZNF280B	ENSG00000275004

TMEM115	ENSG00000126062
ARF4	ENSG00000168374
DENND6A	ENSG00000174839
SLC33A1	ENSG00000169359
MECOM	ENSG00000085276
DCUN1D1	ENSG00000043093
PPP1R2	ENSG00000184203
ITPR1	ENSG00000150995
ATP1B3	ENSG00000069849
CLOCK	ENSG00000134852
PCDH18	ENSG00000189184
FBXW7	ENSG00000109670
PDGFRA	ENSG00000134853
PALLD	ENSG00000129116
STOX2	ENSG00000173320
CLPTM1L	ENSG00000049656
RETREG1	ENSG00000154153
FBXW11	ENSG00000072803
RASA1	ENSG00000145715
DMXL1	ENSG00000172869
MFAP3	ENSG00000037749
PPIL1	ENSG00000137168
CITED2	ENSG00000164442
RGS17	ENSG00000091844
THBS2	ENSG00000186340
FOXF2	ENSG00000137273
PRPF4B	ENSG00000112739
CD2AP	ENSG00000198087
FOXO3	ENSG00000118689
PCMT1	ENSG00000120265
QKI	ENSG00000112531
RAPGEF5	ENSG00000136237
HOXA9	ENSG00000078399
JAZF1	ENSG00000153814

ELMO1	ENSG00000155849
WIPI2	ENSG00000157954
MTURN	ENSG00000180354
7-Sep	ENSG00000122545
STK17A	ENSG00000164543
PPP1R9A	ENSG00000158528
INSIG1	ENSG00000186480
UBE3C	ENSG00000009335
NCALD	ENSG00000104490
HAS2	ENSG00000170961
BAG4	ENSG00000156735
TACC1	ENSG00000147526
GNAQ	ENSG00000156052
VAV2	ENSG00000160293
VLDLR	ENSG00000147852
RECK	ENSG00000122707
TMEM47	ENSG00000147027
LRCH2	ENSG00000130224
SLITRK4	ENSG00000179542
L1CAM	ENSG00000198910
CHIC1	ENSG00000204116
FMR1	ENSG00000102081
